# Let-7b-5p inhibits breast cancer cell growth and metastasis via repression of hexokinase 2-mediated aerobic glycolysis

**DOI:** 10.1038/s41420-023-01412-2

**Published:** 2023-04-05

**Authors:** Ling Li, Xiujuan Zhang, Yanni Lin, Xinxin Ren, Tian Xie, Jing Lin, Shumeng Wu, Qinong Ye

**Affiliations:** 1 Department of Cell Engineering, Beijing Institute of Biotechnology, Beijing, 100850 China; 2 School of Basic Medicine, Shanxi Medical University, Taiyuan, 030000 China; 3 The second hospital of Shanxi Medical University, Taiyuan, 030001 China; 4 Department of Clinical Laboratory, The Fourth Medical Center of PLA General Hospital, Beijing, 100037 China

**Keywords:** Breast cancer, Cancer metabolism

## Abstract

Hexokinase 2 (HK2), a critical rate-limiting enzyme in the glycolytic pathway catalyzing hexose phosphorylation, is overexpressed in multiple human cancers and associated with poor clinicopathological features. Drugs targeting aerobic glycolysis regulators, including HK2, are in development. However, the physiological significance of HK2 inhibitors and mechanisms of HK2 inhibition in cancer cells remain largely unclear. Herein, we show that microRNA-let-7b-5p (let-7b-5p) represses HK2 expression by targeting its 3′-untranslated region. By suppressing HK2-mediated aerobic glycolysis, let-7b-5p restrains breast tumor growth and metastasis both in vitro and in vivo. In patients with breast cancer, let-7b-5p expression is significantly downregulated and is negatively correlated with HK2 expression. Our findings indicate that the let-7b-5p/HK2 axis plays a key role in aerobic glycolysis as well as breast tumor proliferation and metastasis, and targeting this axis is a potential therapeutic strategy for breast cancer.

## Introduction

Breast cancer (BC) registers as the most prevalently occurring malignancy worldwide among women [1]. Despite significant progress in therapy, effective drugs approved for BC remain limited [2]. Therefore, it is crucial to discover new therapeutic targets and biomarkers for BC. Cancer cells exhibit a strong metabolic requirement for energy to sustain their survival and growth [3]. Unlike normal cells, even when the oxygen supply is sufficient, cancer cells predominantly depend on glycolysis for energy, which is known as aerobic glycolysis (Warburg effect) [4, 5]. Aerobic glycolysis, facilitating tumor proliferation with enhanced glucose consumption and lactate concentration, is widely recognized as a hallmark of cancer cells, and targeting this process has been, and continues to be, a focus for therapeutic agent development.

Hexokinase 2 (HK2), which catalyzes the initial rate-limiting and irreversible step of glycolysis reaction, exerts a key role in altered metabolism in various cancers [6–8]. HK2 has been shown to be upregulated in a wide range of human cancers, including hepatocellular carcinoma, breast cancer, gallbladder cancer, colorectal cancer, endometrial carcinoma, osteosarcoma, laryngeal carcinoma, etc., and associated with the clinicopathological characteristics and prognostic factors of cancer patients [6–13]. HK2 promotes cancer cell growth, migration, invasion, and metastasis [14–16]. Recently, HK2-targeted therapy has displayed beneficial effects in suppressing cancer cell growth in vitro and eradicating tumors in animals [7].

MiRNAs (miRNAs) have been reported to influence various biological behaviors in tumors, such as cellular proliferation, differentiation, apoptosis, cell cycle, and so on [17–20]. MiRNA dysregulation might play a significant role in cancer pathogenesis and miRNAs are gradually considered to be potential biomarkers for human cancer diagnosis and treatment [21, 22]. In particular, miRNAs have been shown to exhibit a regulatory effect on glucose metabolism in cancer by inhibiting HK2. For instance, miR-202 inhibits pancreatic cancer cell glycolysis and growth by repressing HK2 expression [23]. MiR-3662 suppresses glucose metabolism, growth, and invasion of hepatocellular carcinoma cells (HCC) by targeting HK2 [24]. MiR-615 functions as a tumor suppressor in osteosarcoma by inhibiting HK2 [12]. However, it is unclear whether miRNAs regulate both tumor proliferation and metastasis through suppression of HK2-mediated aerobic glycolysis.

In the current study, we show that let-7b-5p, a miRNA whose role in modulating cancer glycolysis is unknown, is lowly expressed in BC tissues, and dampens glycolysis in BC cells, subsequently depressing cell proliferation and metastasis both in vitro and in vivo. Mechanistically, HK2 is a new target of let-7b-5p, and let-7b-5p suppresses BC cell glycolysis, proliferation, and metastasis by targeting HK2. In addition, let-7b-5p expression is negatively correlated with HK2 level in patients with BC.

## Results

### Prediction of microRNAs targeting HK2 with clinical significance

**Fig. 1:**
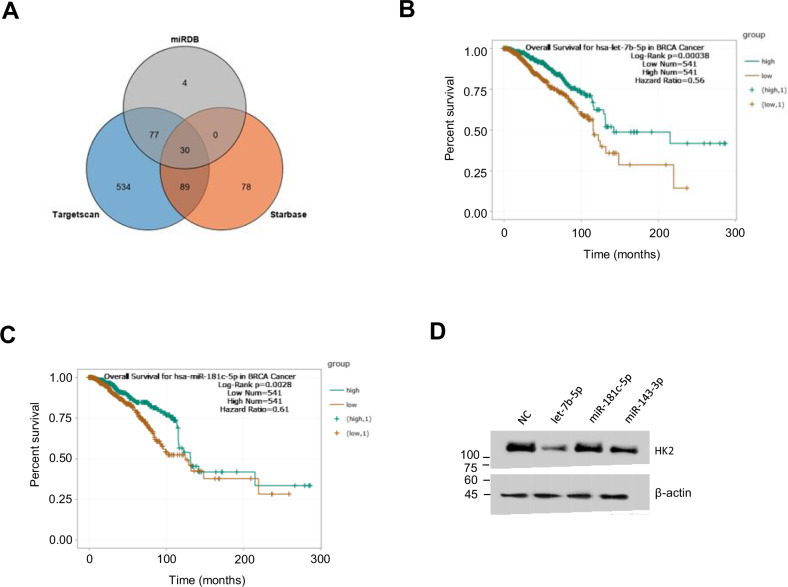
Prediction of microRNAs targeting HK2 with clinical significance. **A** Venn diagram of microRNAs predicted to target HK2 by StarBase, miRDB, and TargetScan Databases. **B**, **C** Overall survival curves for let-7b-5p and miR-181c-5p in 541 BC patients was plotted based on the ENCORI database (https://starbase.sysu.edu.cn/). **D** Western blot detected HK2 expression after transfection of candidate miRNA mimics or negative control (NC) in HEK293T cells (*n* = 3). miR-143-3p acted as a positive control.

Since HK2 is a key enzyme of aerobic glycolysis and performs a vital function in breast cancer, we screened potential miRNAs targeting HK2 using miRDB, TargetScan, and StarBase databases. Thirty miRNAs potentially targeting HK2 were found, including let-7b-5p, let-7c-5p, miR-125a-5p, miR-143-3p, miR-181c-5p, miR-185-5p, miR-493-5p, and so on (Fig. 1A and Table S1). To determine the function of these miRNAs, we investigated their clinical significance in BC by ENCORI database (https://starbase.sysu.edu.cn/), and only found that higher expression of let-7b-5p and miR-181c-5p correlated with longer overall survival (OS) (Fig. 1B, C). Western blot showed that let-7b-5p, miR-181c-5p, and positive control miR-143-3p inhibited HK2 expression in HEK293T cells, with let-7b-5p presenting better inhibition than miR-181c-5p (Fig. 1D). Since the let-7b-5p expression is correlated with clinical prognosis in BC and inhibits HK2 expression, it was chosen for further study.

### HK2 is a target of let-7b-5p in BC cells

**Fig. 2:**
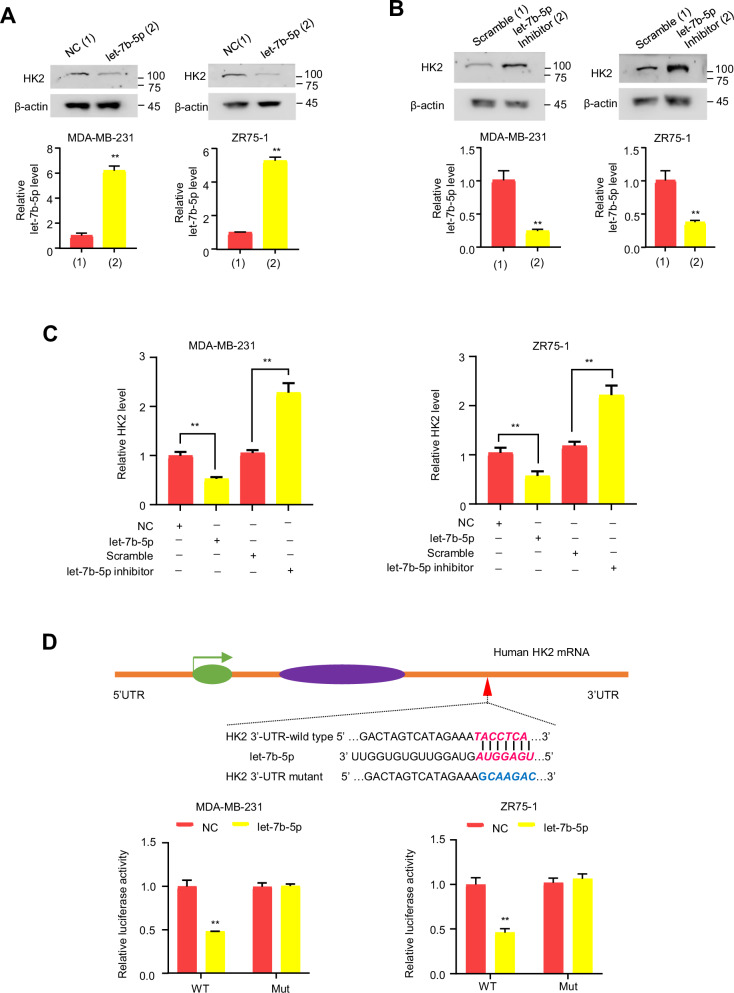
Let-7b-5p targets HK2. **A**, **B** Western blot for HK2 protein expression in indicates BC cells after transfection with let-7b-5p mimic or NC (**A**) or let-7b-5p inhibitor or scramble (**B**) (*n* = 3, mean ± SD). Histograms under western blot show let-7b-5p expression by RT-qPCR (*n* = 3, mean ± SD). **C** RT-qPCR assay of HK2 mRNA expression level in indicated BC cells after transfection with let-7b-5p mimic/inhibitor (*n* = 3, mean ± SD). **D** Dual-luciferase reporter assay of the indicated BC cells after transfection with wild-type or mutated HK2 reporter plus let-7b-5p mimic (*n* = 3, mean ± SD). The top panel shows the putative binding sites between HK2 and let-7b-5p. **p < 0.01 versus corresponding control.

Due to miRNA prediction of HK2 and preliminary confirmation in HEK293T cells by western blot, we carried out western blot for further confirmation. In MDA-MB-231 and ZR75-1 cells, let-7b-5p mimic suppressed HK2 expression (Fig. 2A). On the contrary, let-7b-5p inhibitor led to a dramatic upregulation in HK2 expression (Fig. 2B). Since let-7b-5p has been reported to inhibit HMGA2 expression in head and neck squamous cell carcinoma cells, HCC cells and lung cancer cells [25–27], we chose HMGA2 as a positive control. In MDA-MB-231 and ZR75-1 cells, let-7b-5p mimic suppressed HMGA2 expression (Fig. S1A). On the contrary, the let-7b-5p inhibitor promoted HMGA2 expression (Fig. S1B). These results suggest that the behavior of the let-7b-5p/HMGA2 axis in BC cells may be similar to that in the previously reported cancer cells. To identify how let-7b-5p affects HK2 expression, we examined HK2 mRNA levels, and found that HK2 mRNA levels were downregulated upon let-7b-5p overexpression, while upon let-7b-5p inhibition, they were upregulated (Fig. 2C).

To further explore whether let-7b-5p regulates HK2 expression, a dual-luciferase reporter assay was detected by transfection with HK2 3′-UTR wild-type (WT) or mutated (Mut) luciferase reporter and let-7b-5p in BC cells. Let-7b-5p overexpression diminished HK2 3′-UTR WT luciferase activity, but not HK2 3′-UTR Mut luciferase activity (Fig. 2D). The results indicate that let-7b-5p targets HK2 3′-UTR to inhibit its expression in BC cells.

### Let-7b-5p depresses proliferation, migration, and invasion of BC cells by targeting HK2

**Fig. 3:**
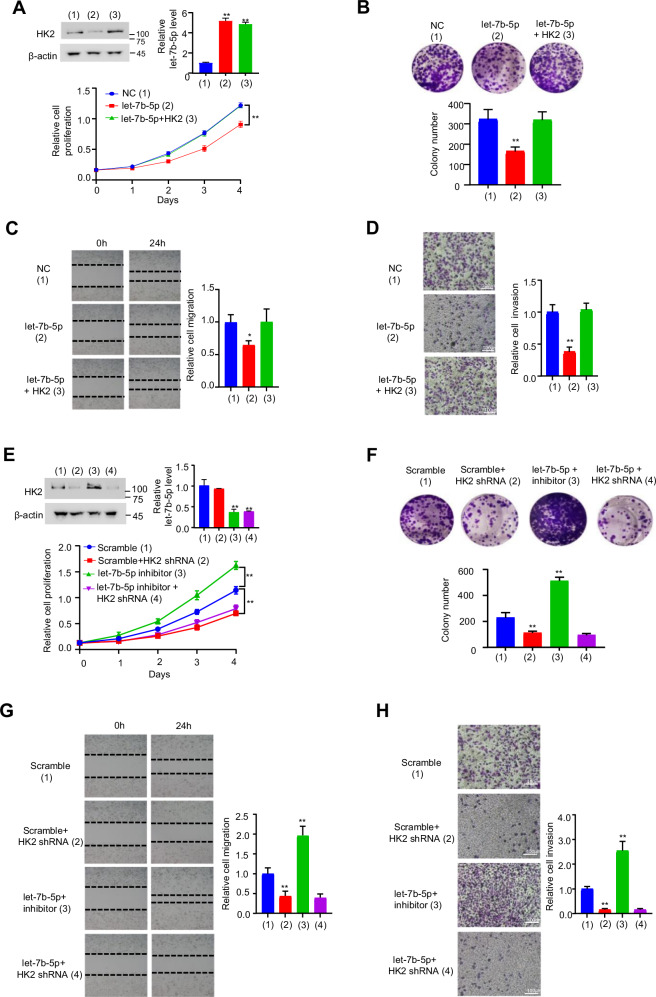
Let-7b-5p dampens BC cell growth, migration, and invasion via repression of HK2 expression. **A** Growth curve was analyzed by CCK-8 Kit after MDA-MB-231 cells were transfected with NC, let-7b-5p mimic, or let-7b-5p mimic plus HK2 plasmid (*n* = 3, mean ± SD). Western blot and RT-qPCR showed HK2 and let-7b-5p expression, respectively. **B** Colony formation analysis of MDA-MB-231 cells after the transfection as in (**A**). Histograms display the colony number (*n* = 3, mean ± SD). **C**, **D** Scratch test (**C**) and transwell assay (**D**) of MDA-MB-231 cells after the transfection as in (**A**). Histograms display relative cell migration or invasion (*n* = 3, mean ± SD). **E**, **F** Control or HK2 shRNA MDA-MB-231 cells with the transfection of let-7b-5p inhibitor or scramble were analyzed as in (**A**) and (**B**) (*n* = 3, mean ± SD). **G**, **H** Scratch test (**G**) and transwell assay (**H**) of control or HK2 shRNA MDA-MB-231 cells with the transfection as in (**E**, **F**) (*n* = 3, mean ± SD). Scale bar, 100 μm. *p < 0.05, **p < 0.01 versus corresponding control.

TCGA dataset showed that let-7b-5p was downregulated in BC [28], suggesting that let-7b-5p may act as a tumor suppressor in BC. However, the biological role of let-7b-5p in BC is still unknown. Since let-7b-5p targets HK2 and HK2 promotes BC cell proliferation, migration, and invasion, we tested if let-7b-5p exerts a role on BC and its function relies on HK2. We found that let-7b-5p overexpression in MDA-MB-231 and ZR75-1 cells reduced cell proliferation, migration, and invasion, while the effects could be reversed by HK2 reexpression (Fig. 3A–D and Fig. S2A–D). Moreover, let-7b-5p inhibitor accelerated the proliferation, migration, and invasion of BC cells, and HK2 knockdown abrogated this effect (Fig. 3E–H and Fig. S2E–H). The results reveal that let-7b-5p represses proliferation, migration and invasion of BC cells by HK2 inhibition.

### Let-7b-5p impairs glycolysis by inhibiting HK2 in BC cells

**Fig. 4:**
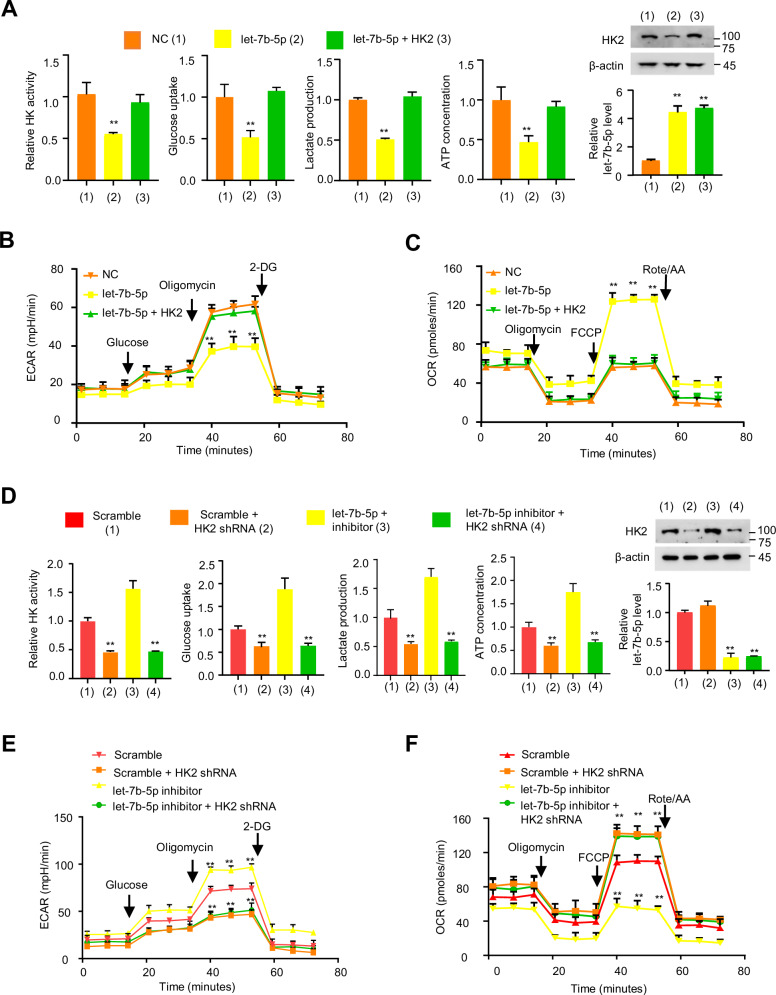
Let-7b-5p impairs glycolysis by suppression of HK2 expression in BC cells. **A** HK activity, glucose uptake, lactate production, and ATP concentration assays of MDA-MB-231 cells after transfection with NC, let-7b-5p mimic or let-7b-5p mimic plus HK2 plasmid (*n* = 3, mean ± SD). Western blot and RT-qPCR analysis reveal HK2 and let-7b-5p expression, respectively (*n* = 3, mean ± SD). **B**, **C** ECAR (**B**) and OCR (**C**) were assayed after MDA-MB-231 cells were transfected as in (**A**) (*n* = 4, mean ± SD). **D** Control or HK2 shRNA MDA-MB-231 cells with transfection of let-7b-5p inhibitor or scramble were detected as in (**A**) (*n* = 3, mean ± SD). **E**, **F** ECAR (**E**) and OCR (**F**) detection of control or HK2 shRNA MDA-MB-231 cells after transfection as in (**D**) (*n* = 4, mean ± SD). **p < 0.01 versus corresponding control.

Considering that aerobic glycolysis is important for influencing BC cell progression by HK2, and let-7b-5p inhibits HK2 expression, we then explored whether let-7b-5p regulates glycolysis via HK2. We examined the role of let-7b-5p on hexokinase (HK) enzyme activity, glucose uptake, lactate production, and ATP concentration in MDA-MB-231 and ZR75-1 cells (Fig. 4A and Fig. S3A). Let-7b-5p mimic decreased the HK activity, glucose uptake, lactate level, and ATP concentration, and HK2 reexpression rescued these influences. Furthermore, let-7b-5p mimics decreased extracellular acidification (ECAR) and increased oxygen consumption (OCR), and HK2 reexpression rescued these effects (Fig. 4B, C and Fig. S3B, C). In addition, the let-7b-5p inhibitor greatly increased glycolytic phenotype, and the knockdown of HK2 undermined these effects (Fig. 4D–F and Fig. S3D–F). Accordingly, these findings indicate that let-7b-5p inhibits glycolysis by repressing HK2 in BC cells.

### Let-7b-5p regulates the proliferation, migration, and invasion of BC cells by aerobic glycolysis

**Fig. 5:**
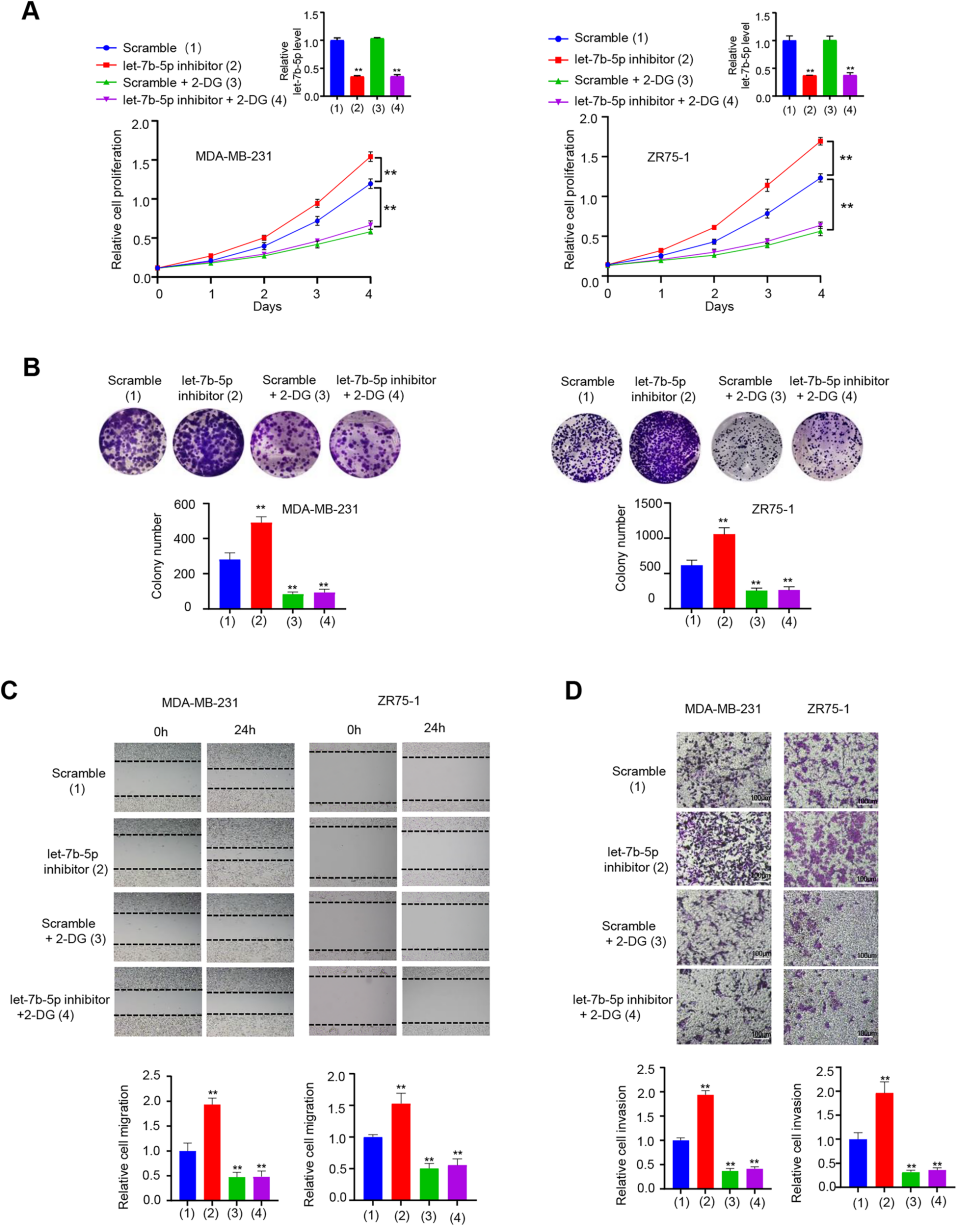
Let-7b-5p relies on glycolysis to regulate BC cell proliferation and migration. **A** Growth curve of the indicated BC cells were assayed by CCK-8-kit after the transfection of let-7b-5p inhibitor or scramble and treatment of 2.5 mM 2-DG (*n* = 3, mean ± SD). RT-qPCR analyzed let-7b-5p expression (*n* = 3, mean ± SD). **B** Colony formation assay of the indicated cells after transfection as in (**A**). Histograms display the colony number (*n* = 3, mean ± SD). **C**, **D** Scratch test (**C**) and transwell assay (**D**) of the indicated cells after the transfection as in (**A**). Histograms display relative cell migration or invasion (*n* = 3, mean ± SD). **p < 0.01 versus corresponding control.

Considering let-7b-5p modulates glycolysis as well as proliferation, migration, and invasion of BC cells via HK2, we used glycolysis inhibitor 2-Deoxy-D-glucose (2-DG) to investigate whether let-7b-5p/HK2 axis influences these phenotypes by glycolysis. In MDA-MB-231 and ZR75-1 cells, the proliferation, migration, and invasion enhancement mediated via let-7b-5p inhibitor was reverted by 2-DG (Fig. 5A–D).

### Let-7b-5p/HK2 axis regulates in vivo glycolysis, tumorigenesis, and metastasis in BC

**Fig. 6:**
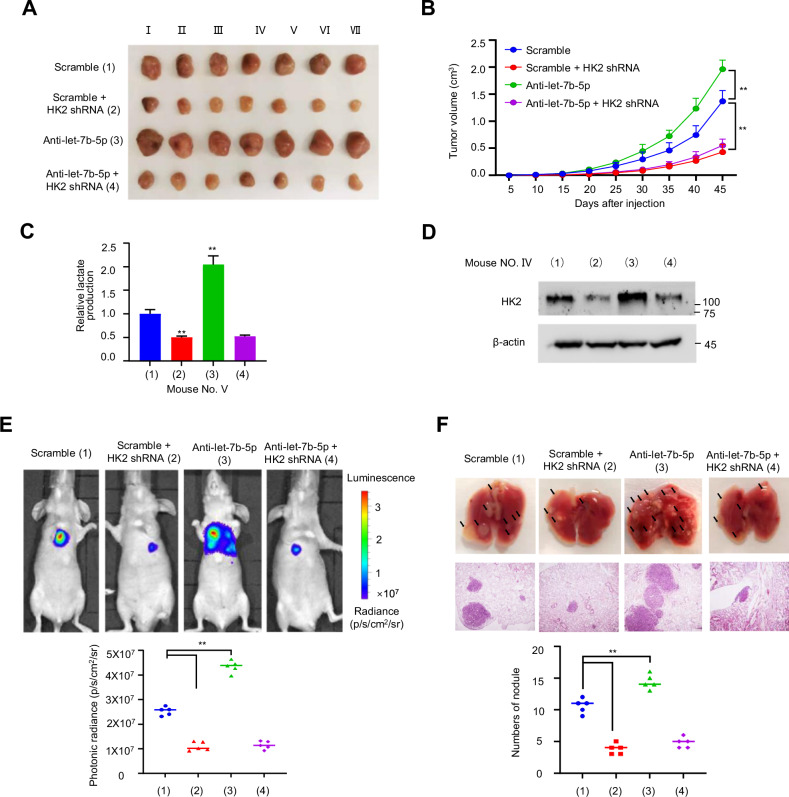
Let-7b-5p/HK2 axis modulates glycolysis, tumor proliferation, and metastasis in mice. **A**, **B** Control or HK2 shRNA MDA-MB-231 cells were treated with anti-let-7b-5p (antagomiR-let-7b-5p) or scramble (antagomiR-NC) and injected into nude mice. Tumor size was detected at indicated times, and a proliferation curve was drew (*n* = 7, mean ± SD). **C** Lactic acid concentration of representative tumors from (**A**) was assayed (*n* = 3, mean ± SD). **D** Western blot of HK2 expression in representative tumors from (**A**). **E** Representative bioluminescence images were collected from lung metastasis models by injecting indicated MDA-MB-231 cells into the tail vein of nude mice (*n* = 5). The bioluminescence signal is presented by an overlaid false-color image with the signal intensity indicated by the scale. **F** Representative photographs of lungs from **E** and H&E staining of lung tissue sections. The scatter diagram showed the number of metastatic nodules.

To verify the in vivo effect of the let-7b-5p/HK2 axis, we established nude mouse xenograft tumor models of BC. As expected, the let-7b-5p inhibitor significantly enhanced the breast tumor growth of MDA-MB-231 cells, while HK2 knockdown dramatically resisted the growth (Fig. 6A, B). Importantly, let-7b-5p inhibitor-mediated enhancement of tumor growth was abrogated when HK2 was knocked down, revealing that let-7b-5p modulates breast tumor growth by HK2. Further tumor lactate analysis verified that let-7b-5p regulated lactate production via HK2 (Fig. 6C, D). Moreover, let-7b-5p inhibitor promoted lung metastasis of breast tumors, whereas HK2 knockdown blocked this effect (Fig. 6E). Furthermore, let-7b-5p inhibitor-mediated lung metastasis was abrogated when HK2 was knocked down. The metastasis foci were confirmed via histologic analysis on the lungs (Fig. 6F). These data display that let-7b-5p depresses breast tumorigenesis and metastasis in vivo via HK2.

### Let-7b-5p is negatively correlated with HK2 in patients with BC

**Fig. 7:**
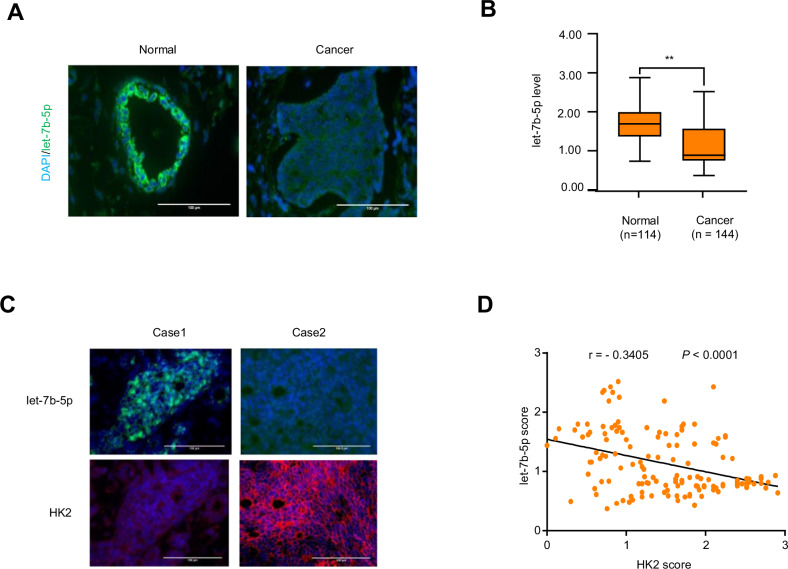
Correlation analysis between let-7b-5p and HK2 in patients with breast cancer. **A** Immunofluorescence staining of let-7b-5p in 114 normal samples and 144 BC samples. Scale bar, 100 μm. **B** Let-7b-5p expression was analyzed between 114 normal and 144 BC tissues by Mann–Whitney U test. **C** Representative immunofluorescence imaging o of 144 BC patients. HK2 expression were detected by IHC, and let-7b-5p expression were detected by miRNA FISH. Scale bar, 100 μm. **D** Correlation analysis of let-7b-5p and HK2 expression in 144 BC patients.

To assess the clinical association between let-7b-5p and HK2, let-7b-5p expression was detected in breast cancer and normal tissues. Intriguingly, let-7b-5p was downregulated in BC samples compared with normal samples (Fig. 7A, B). The specificity of the let-7b-5p probe was verified by miRNA fluorescence in situ hybridization (FISH) (Fig. S4A). Moreover, HK2 expression was detected by immunocytochemistry (IHC) and let-7b-5p expression was by miRNA FISH in BC tissues. In agreement with the phenomenon that let-7b-5p represses HK2 in cultured cells, let-7b-5p level negatively correlated with HK2 level (Fig. 7C, D). We identified the specificity of the HK2 antibody using IHC of BC tissues (Fig. S4B). Accordingly, these data strongly support that the let-7b-5p/HK2 axis plays critical pathological roles in breast cancer.

## Discussion

Metabolic reprogramming, especially for aerobic glycolysis (Warburg effect), is an emerging hallmark of cancer [4, 5]. Targeting metabolic pathway is increasingly recognized as an efficient way to control tumor growth and enhance anti-cancer therapy. Metabolic enzymes in the pathway have been paid much attention as targets for anti-cancer treatments. As a result, researchers are trying hard to find new drugs that target metabolic enzymes by blocking glucose metabolism.

HK2, one of the key metabolic enzymes, catalyzes the irreversible rate-limiting step of glycolysis and accelerates aerobic glycolysis and cancer progression. High expression of HK2 has been shown associated with poor clinical prognosis in patients with cancer [29]. Therefore, developing HK2 inhibitors is significant. Currently, some miRNAs have been reported as inhibitors of HK2. MiR-216b potentiates breast cancer cell autophagy and apoptosis in vitro by targeting HK2 through the mTOR signaling pathway [30]. Resibufogenin regulates the miR-143-3p/HK2 axis to inhibit tumor growth and glycolysis in breast cancer [31]. MiR-3662 and miR-125a act as suppressors for glucose metabolism by HK2 inhibition, and suppress cell proliferation, invasion, or apoptosis in hepatocellular carcinoma cells in vitro [24, 32]. However, the significance of the physiology and pathology of these natural miRNAs molecules is unclear. Our research found that let-7b-5p is a novel inhibitor of HK2, inhibits HK enzyme activity, glucose uptake, lactate level, and ATP concentration, and leads to conversion from aerobic glycolysis to mitochondrial respiration via repressing HK2 in BC cells. HK2 has two isoforms (NM_000189.5 and NM_001371525.1), which share the same 3’-UTR sequence. As let-7b-5p inhibits HK2 expression by targeting its 3’-UTR, it is conceivable that let-7b-5p represses both HK2 isoforms. Let-7b-5p depresses BC proliferation and lung metastasis by suppression of HK2-mediated aerobic glycolysis. Furthermore, let-7b-5p negatively correlates with HK2 in BC tissues. Therefore, these data illustrate the let-7b-5p significance for physiology and pathology in modulating HK2-mediated aerobic glycolysis as well as tumorigenesis and lung metastasis. Upregulation of let-7b-5p could be a promising approach for BC therapy with HK2 overexpression.

Although we show that let-7b-5p regulates BC cell migration and invasion by targeting HK2, we cannot exclude the possibility that it may target other RNAs. It has been reported that let-7b-5p inhibits migration, invasion, and EMT by targeting HMGA2 in head and neck squamous cell carcinoma and HCC cells [25, 26]. We also showed that let-7b-5p could suppress HMGA2 expression in BC cells. Since HMGA2 has been reported to influence cell growth, migration, and invasion in BC cells [33], HMGA2 may be another potential target of let-7b-5p that is involved in these biological processes.

Recently, let-7b-5p has been identified to have different roles in regulating tumorigenesis and cancer progression. As a tumor suppressor, let-7b-5p inhibits growth and apoptosis by targeting IGF1R in multiple myeloma [34]; let-7b-5p suppresses proliferation and motility by negatively modulating KIAA1377 in squamous cell carcinoma cells [35]. The anti-cancer roles were also confirmed in other cancers, such as human glioma and gastric cancer [36, 37]. As a tumor-promoting factor, let-7b-5p is overexpressed in ovarian cancer, and its silence dampens ovarian cancer cell proliferation [38]. Suppression of let-7b-5p is conducive to an anti-tumorigenic macrophage phenotype in prostate cancer by SOCS1/STAT pathway [39]. The findings show that let-7b-5p plays a tissue-specific role in different types of cancer. Previous research have presented that let-7b-5p was downregulated in BC [28] and overexpression of let-7b-5p was associated with better OS and disease-free survival (DFS) in all breast cancer cases [40] by TCGA dataset analysis. However, the influence of let-7b-5p on the Warburg effect and its mechanism in regulating breast cancer is still unclear. We showed that let-7b-5p suppresses not only aerobic glycolysis but also the growth and metastasis of breast tumors by inhibiting HK2-mediated glycolysis. Therefore, our research presents a molecular explanation which links the anti-cancer effect of let-7b-5p in inhibiting breast tumor progression with its ability to dampen glycolysis. In addition, let-7b-5p associates glycolysis with breast tumor proliferation and lung metastasis in vivo.

Estrogen receptor (ER) and breast-cancer susceptibility gene (BRCA) are widely recognized as important markers for BC. ER is not only a powerful predictive and prognostic marker but also a valuable target for the treatment of hormone-dependent breast cancer. BRCA, which includes BRCA1 and BRCA2, is a critical tumor suppressor gene for BC. Mutations in BRCA can cause chromosomal instability, promote cell proliferation, and hinder normal cell differentiation, leading to the development of BC. Recent discoveries have indicated that there are some correlations between such BC markers, let-7b and HK2. Let-7b has been shown to inhibit the expression of ER-α, which is inversely correlated with let-7b in BC tissues [41, 42]. Estradiol (E2) treatment has been found to promote HK2 expression in paclitaxel-resistant BC cells [43]. Dysregulation of let-7b has also been observed in BRCA2 germ-line mutation carriers between invasive breast cancer and asymptomatic normal breast tissue [44]. Furthermore, BRCA1 has been found to repress HK2 expression, reducing glycolysis and attenuating BC cell migration [45].

Overall, our study demonstrates that let-7b-5p dampens BC cell growth and metastasis in vitro and in vivo by suppressing glycolysis via inhibiting the expression of HK2. Let-7b-5p negatively correlates with HK2 in patients with breast cancer. These results verify the significance of the let-7b-5p/HK2 axis in aerobic glycolysis as well as breast tumorigenesis and progression. Therefore, let-7b-5p could be valuable for treating HK2-overexpressing breast cancer patients.

## Materials and methods

### Cell culture

MDA-MB-231, ZR75-1, and HEK293T cell lines were obtained from American Type Culture Collection (ATCC). MDA-MB-231 cell line labeled with firefly luciferase was a gift from Professor Yongfeng Shang. All cells were cultured in DMEM (Gibco) appended to 10% FBS (Everygreen) and 100 μg/ml penicillin and streptomycin (Biomed) at 37˚C with 5% CO_2_.

### RNA oligonucleotides, plasmids, lentivirus, regents

Let-7b-5p mimic/inhibitor was purchased from GenePharma. Wild-type and mutated sequences of the HK2 3′-UTR were inserted into a pcDNA3-luciferase expression vector, generating HK2 3′-UTR WT and HK2 3′-UTR MUT, respectively. HK2 expression vector was constructed by inserting PCR-amplified fragments into pcDNA3 (Invitrogen). HK2 shRNA stable cell line was established by lentiviral transduction using pSIH-H1-Puro (System Biosciences) carrying HK2 shRNA. The target sequence of HK2 shRNA was ATAAGCTACAAATCAAAGA. Stable cells that were infected with lentiviruses were screened using puromycin. Reagents for miRNAs and plasmids transfection were, respectively, Lipofectamine RNAiMAX and Lipofectamine 3000 (Invitrogen). Anti-HK2 antibody was obtained from Cell Signaling Technology and an anti-β-actin antibody was obtained from Santa Cruz Biotechnology.

### Quantitative real-time PCR (RT-qPCR)

Total RNA, including mRNA and miRNA, was extracted with TRIzol reagent (Invitrogen). miRcute Plus miRNA First-Strand cDNA Kit (Tiangen) was used to transcribe miRNA into cDNA. RT-qPCR analysis was determined with 2 × Taq Pro Universal SYBR qPCR Master Mix (Vazyme) using the BioRad CFX96. The relative fold expression of the targets was normalized to U6 or β-actin (endogenous control) and calculated by the 2^−∆∆Ct^ method. Primer sequences used are listed in Table S2.

### Luciferase reporter assay

Cells seeded in a 24-well plate were co-transfected with negative control (NC) or let-7b-5p mimic, in combination with luciferase reporters HK2 3′-UTR WT/ Mut and pRL-TK (internal control) using Lipofectamine 3000. Luciferase activities analysis were performed 48 h later following the manufacturer’s instruction (Promega).

### Cell proliferation, migration, and invasion assays

Cell proliferation was performed using a CCK-8 kit and colony formation assay. Cell migration was examined by scratch test. Cell invasion was assessed by transwell assay with Matrigel Invasion Chambers. These assays were conducted according to the methods described previously [46].

### Glycolytic phenotype assay

Hexokinase Colorimetric Assay Kit, Glucose Uptake Colorimetric Assay kit, ATP Colorimetric Assay kit and Lactate Assay Kit II were purchased from Biovision and used to detect HK activity, glucose uptake, ATP, and lactate production, respectively. These assays were detected following the manufacturer’s protocols as described previously [47].

### ECAR and OCR assays

ECAR were examined by Seahorse XF Glycolysis Stress Test Kit and OCR were examined by Seahorse XF Cell Mito Stress Test Kit (Agilent Technologies). Samples were detected via Seahorse XF^e^ 96 Extracellular Flux Analyzer (Seahorse Bioscience). The assays were performed referring to manufacturer-provided protocols as described previously [48].

### Tumorigenesis and metastasis in nude mice

Animal experiments were approved by the Institutional Animal Care Committee of the Beijing Institute of Biotechnology. For tumorigenesis analysis, ten million MDA-MB-231 cells stably carrying control or HK2 shRNA treated with 1 μmol antagomiR-let-7b-5p (anti-let-7b-5p) or antagomiR-NC (scramble) for 3 days were subcutaneously inoculated into female BALB/c nude mice (6 to 8 weeks old) which were randomly selected seven into each group without blinding. Tumor size was detected by vernier caliper every 5 days and tumor volume was calculated as the formula: (length × width^2^)/2. After 45 days, the mice were sacrificed and dissected tumors were imaged, and then frozen in liquid nitrogen for further study.

For the metastasis experiment, one million of these treated MDA-MB-231 cells were injected into female BALB/c nude mouse (*n* = 5/group) by lateral tail vein [47]. Thirty days later, these mice images were captured by the IVIS200 imaging system (Xenogen Corporation) and metastatic foci of lung tissues was analyzed by H&E staining.

### Clinical samples, miRNA FISH, and IHC

Samples of 144 human breast cancer and 114 normal tissues were obtained from the PLA General Hospital, with the informed consent of patients and approval of the Institutional Review Committees of the Chinese PLA General Hospital. The expression level of let-7b-5p was determined following miRNA FISH instructions (Exonbio). Let-7b-5p probe (FITC labeled) sequence was AACCACACAACCTACTACCTCA. The scramble probe (negative control) sequence was GTGTAACACGTCTATACGCCCA. The level of HK2 expression was determined by IHC and cyanine 3 system (K1051, APExBIO). Anti-HK2 antibody (Cell Signaling Technology) was used as the primary antibody. IHC of specimens was analyzed as previously described [49]. The fluorescence intensity was examined using a microscope (BX53F; Olympus, Tokyo, Japan). The let-7b-5p or HK2 score was calculated by multiplying staining intensity (1, low; 2, medium; 3, strong) by stained cells percentage (0–100%).

### Statistical analysis

Statistical analyses were processed with GraphPad Prism 7 software. Comparisons among multiple groups were analyzed by One-way ANOVA. Means between the two groups were compared by Student’s *t*-test. Correlation analysis between HK2 and let-7b-5p expression was represented using Spearman rank correlation. *P* < 0.05 was considered statistically significant. All experiments in vitro were performed in triplicates.

## Supplementary information

let-7b-5p-S

Table S1. The intersection of miRNAs targeing HK2 in StarBase, miRDB and TargetScan

Table S2. Primers used for RT-qPCR

Western Blots Figures

## Data Availability

All data generated or analyzed presented in this study are included in the article and its supplementary files.
