# Correction: Let-7b-5p inhibits breast cancer cell growth and metastasis via repression of hexokinase 2-mediated aerobic glycolysis

**DOI:** 10.1038/s41420-026-03069-z

**Published:** 2026-04-22

**Authors:** Ling Li, Xiujuan Zhang, Yanni Lin, Xinxin Ren, Tian Xie, Jing Lin, Shumeng Wu, Qinong Ye

**Affiliations:** 1 Department of Cell Engineering, Beijing Institute of Biotechnology, Beijing, 100850 China; 2 School of Basic Medicine, Shanxi Medical University, Taiyuan, 030000 China; 3 The second hospital of Shanxi Medical University, Taiyuan, 030001 China; 4 Department of Clinical Laboratory, The Fourth Medical Center of PLA General Hospital, Beijing, 100037 China

Correction to: *Cell Death Discovery* 10.1038/s41420-023-01412-2, published online 05 April 2023

Upon a thorough review of the published content, we recently identified mistakes in Fig. 5C, Fig. 5D and Fig. S2D due to oversights during the figure preparation process. Specifically, we mistakenly used the same Wound healing in MDA-MB-231 and ZR75-1 cells in Fig. 5C. The transwell for (3) and (4) in ZR75-1 cells in Fig. 5D was mistakenly placed. Additionally, the transwell for (1) was mistakenly placed in Fig. S2D. Results section are replaced as below. These corrections do not affect the results and conclusions of this study. We deeply apologize for any confusion or inconvenience that this may have caused.

**Original file for figure 5** 
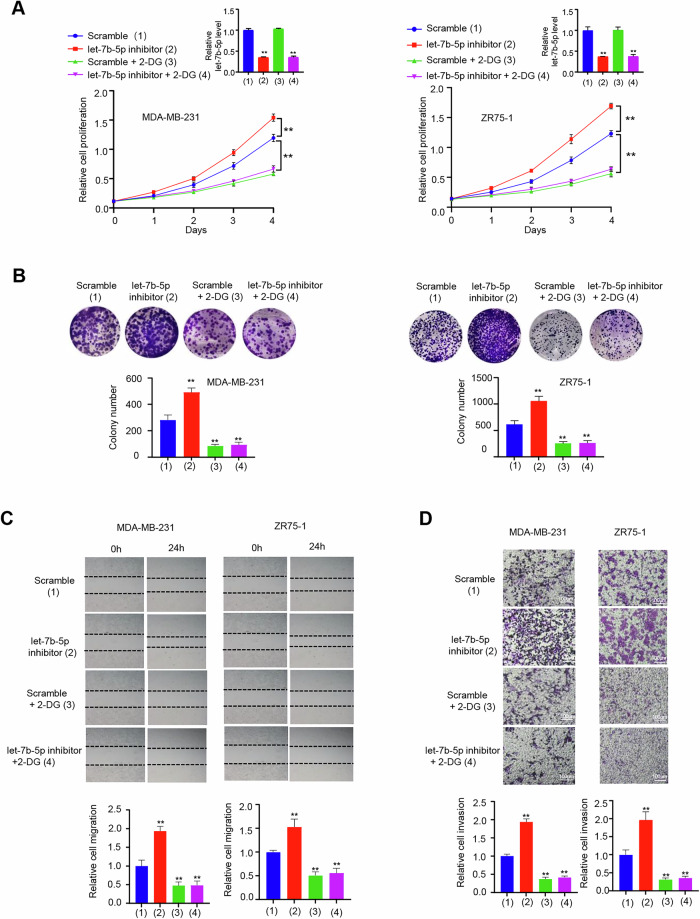


**Amended file for figure 5** 
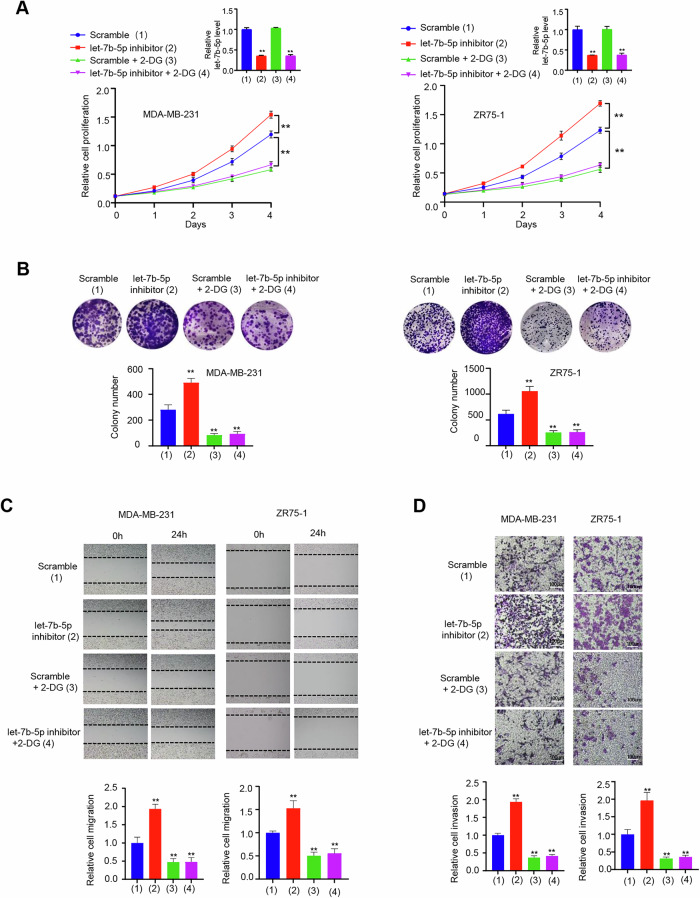


**Original file for figure S2** 
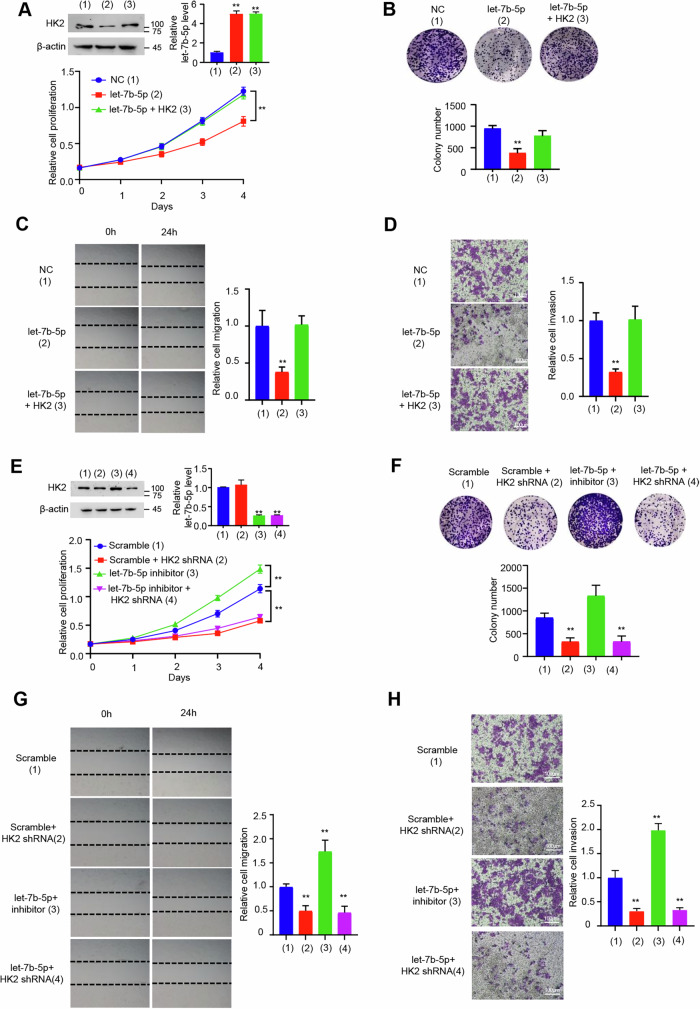


**Amended file for figure S2** 
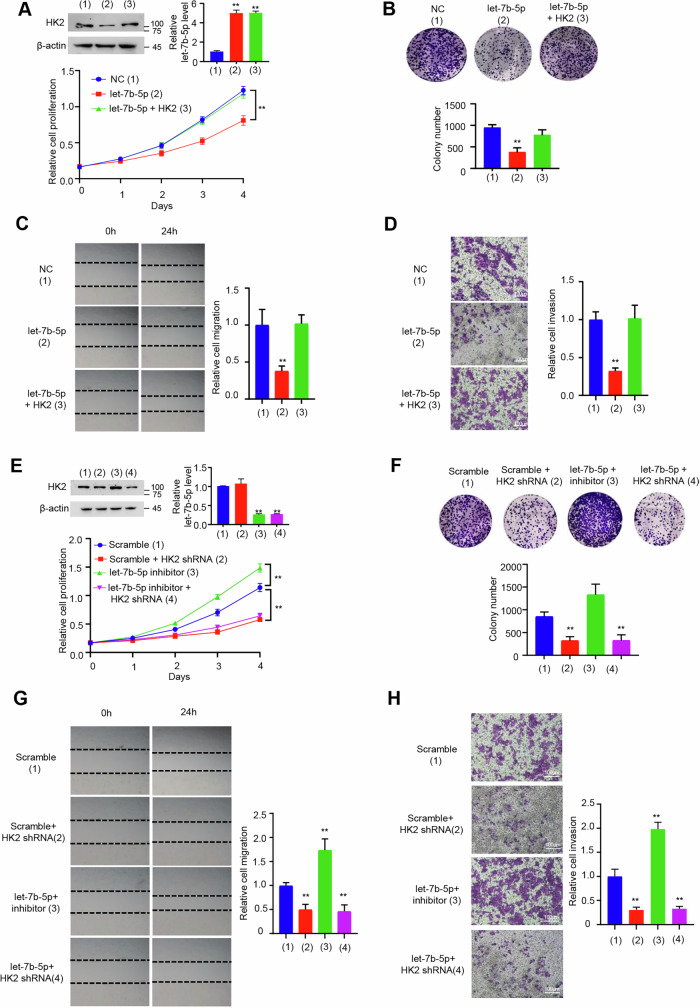


The original article has been corrected.

## Supplementary information

Amended file for figure S2

Original file for figure S2

