# Supplementary material for: Let-7b-5p inhibits breast cancer cell growth and metastasis via repression of hexokinase 2-mediated aerobic glycolysis
Source: Cell Death Discov. 2023 Apr 5;9:114. doi: 10.1038/s41420-023-01412-2 (PMC10076263; doi:10.1038/s41420-023-01412-2)
Supplement: Supplementary file 1 — let-7b-5p-S [file 41420_2023_1412_MOESM1_ESM.pdf]

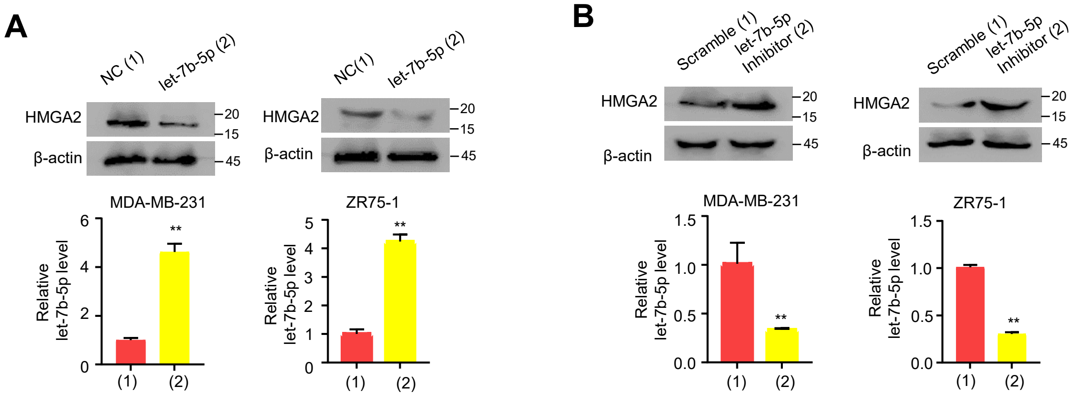

Fig. S1 Let-7b-5p targets HMGA2. **A, B** Western blot for HMGA2 protein expression in indicates BC cells after transfection with let-7b-5p mimic or NC (**A**), or let-7b-5p inhibitor or scramble (**B**) (n = 3, mean ± SD). Histograms under western blot show let-7b-5p expression by RT-qPCR (n = 3, mean ± SD). **p < 0.01 versus corresponding control.

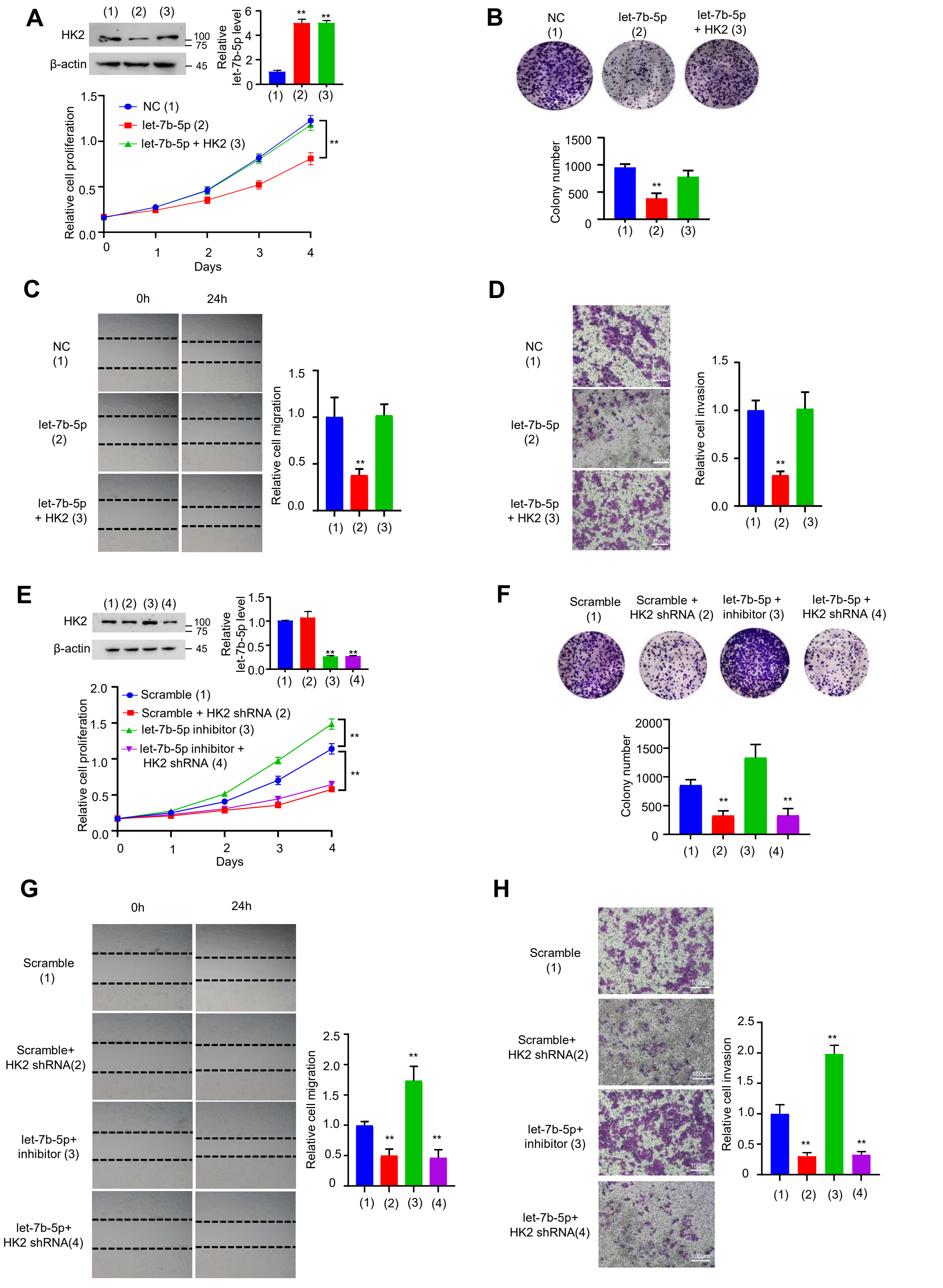

Fig. S2 Let-7b-5p suppresses proliferation, migration and invasion through inhibition of HK2 expression in ZR75-1 cells. **A** Proliferation curve was analyzed by CCK-8 Kit after ZR75-1 cells were transfected with NC, let-7b-5p mimic or let-7b-5p mimic plus HK2 plasmid (n = 3, mean ± SD). Western blot and RT-qPCR showed HK2 and let-7b-5p expression respectively. **B** Colony formation analysis of ZR75-1 cells after the transfection as in (A). Histograms display the colony number (n = 3, mean ± SD). **C, D** Scratch test (**C**) and transwell assay (**D**) of ZR75-1 cells after the transfection as in (A). Histograms display relative cell migration or invasion (n = 3, mean ± SD). **E, F** Control or HK2 shRNA ZR75-1 cells with the transfection of let-7b-5p inhibitor or scramble were analyzed as in (A) and (B) (n = 3, mean ± SD). **G, H** Scratch test (**G**) and transwell assay (**H**) of control or HK2 shRNA ZR75-1 cells with the transfection as in (E, F) (n = 3, mean ± SD). Scale bar, 100 μm. *p < 0.05, **p < 0.01 versus corresponding control.

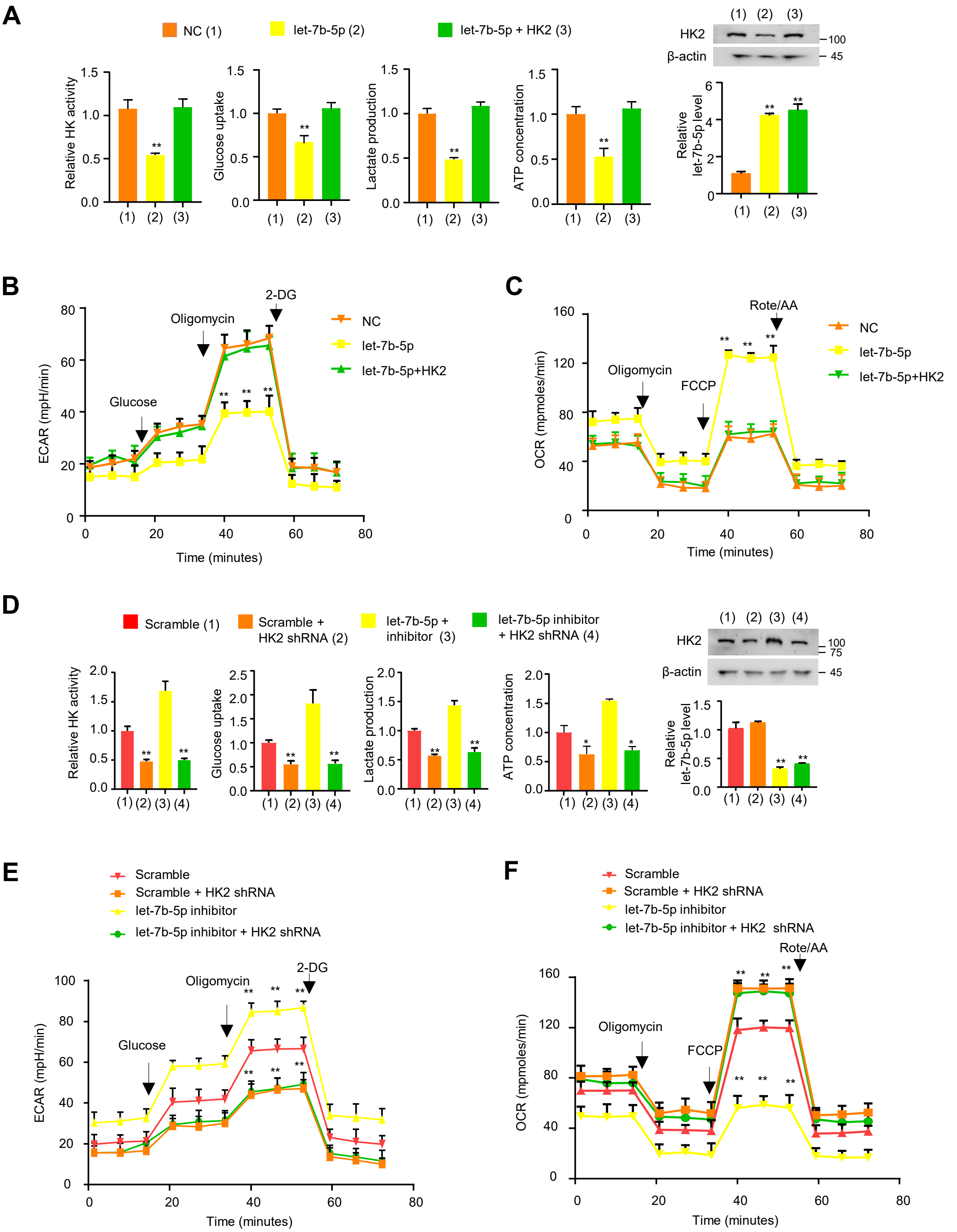

Fig. S3 Let-7b-5p dampens glycolysis via inhibition of HK2 expression in ZR75-1 cells. A ZR75-1 cells were transfected with NC, let-7b-5p mimics or let-7b-5p mimics plus HK2. HK activity, glucose uptake and the production of lactate and ATP were determined (n = 3, mean ± SD). Representative immunoblot reveals HK2 expression. qRT-PCR analysis indicates let-7b-5p expression. **B, C** ZR75-1 cells were transfected as in (A), and ECAR (**B**) and OCR (**C**) were then determined (n = 4, mean ± SD). **D** Control and HK2 shRNA ZR75-1 cells were transfected with scramble or let-7b-5p inhibitor and analyzed as in (A) (n = 3, mean ± SD). . **E, F** ECAR (**E**) and OCR (**F**) assays of control and HK2 shRNA ZR75-1 cells after transfection as in (D) (n = 4, mean ± SD). $^{**}P < 0.01$ versus corresponding control.

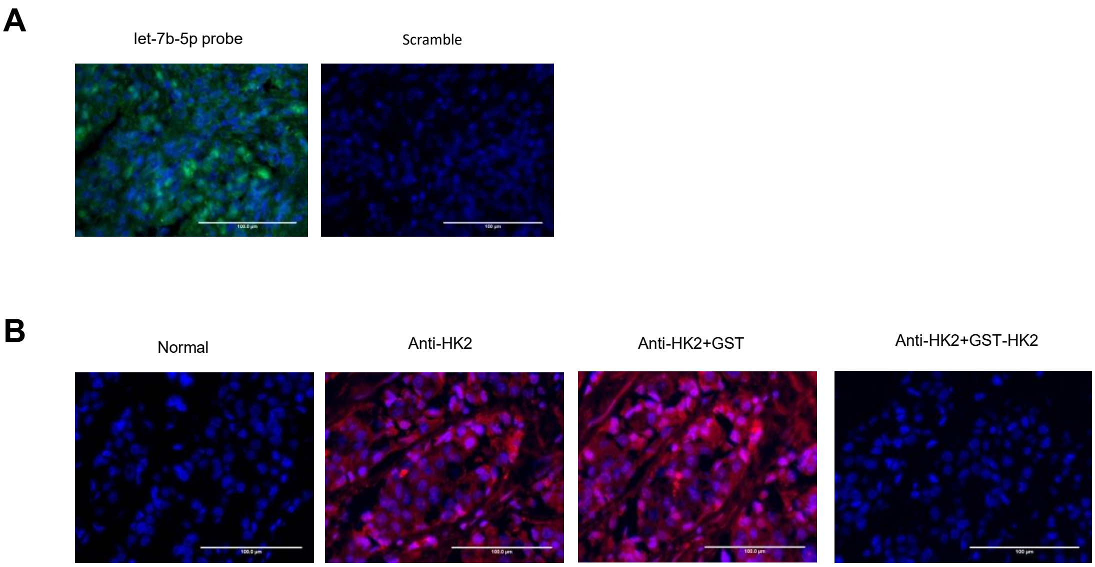

Fig. S4 Specificity validation of let-7b-5p probe and HK2 antibody. A FISH of breast cancer samples with let-7b-5p probe or scramble probe. Scale bar, 100 um. **B** Immunohistochemical staining of breast cancer samples incubated with normal IgG or anti-HK2. To validate antibody specificity, the anti-HK2 was pre-incubated with recombinant GST-HK2 protein or GST for 1 h prior to applying to tissue. Scale bar, 100 um.
