# Supplementary material for: Let-7b-5p inhibits breast cancer cell growth and metastasis via repression of hexokinase 2-mediated aerobic glycolysis
Source: Cell Death Discov. 2023 Apr 5;9:114. doi: 10.1038/s41420-023-01412-2 (PMC10076263; doi:10.1038/s41420-023-01412-2)
Supplement: Supplementary file 3 — Table S2. Primers used for RT-qPCR [file 41420_2023_1412_MOESM3_ESM.docx]

**Table S2. Primers used for RT-qPCR**

| **Gene** | **Forward (5’→3’)** | **Reverse (5’→3’)** |
| --- | --- | --- |
| Let-7b-5p  U6 ^[1]^  HK2 ^[1]^  β-actin ^[2]^ | TGAGGTAGTAGGTTGTGTGG CGCGCTTCGGCAGCACATATACT  GCCATCCTGCAACACTTAGGGCTTGAG  TCGTGCGTGACATTAAGGAG | CAGTGCGTGTCGTGGAGT ACGCTTCACGAATTTGCGTGTC  GTGAGGATGTAGCTTGTAGAGGGTCCC  ATGCCAGGGTACATGGTGGT |

**References:**

[1] Li L, Liang Y, Kang L, Liu Y, Gao S, Chen S, et al. Transcriptional regulation of the Warburg effect in cancer by SIX1. Cancer Cell. 2018;33:368-85.

[2] Shi Y, Zhang Y, Ran F, Liu J, Lin J, Hao X, et al. Let-7a-5p inhibits triple-negative breast tumor growth and metastasis through GLUT12-mediated Warburg effect. Cancer Lett. 2020;495:53-65.
