# Supplementary figures and images for: Let-7b-5p inhibits breast cancer cell growth and metastasis via repression of hexokinase 2-mediated aerobic glycolysis
Source: Cell Death Discov. 2023 Apr 5;9:114. doi: 10.1038/s41420-023-01412-2 (PMC10076263; doi:10.1038/s41420-023-01412-2)

Fig. 1

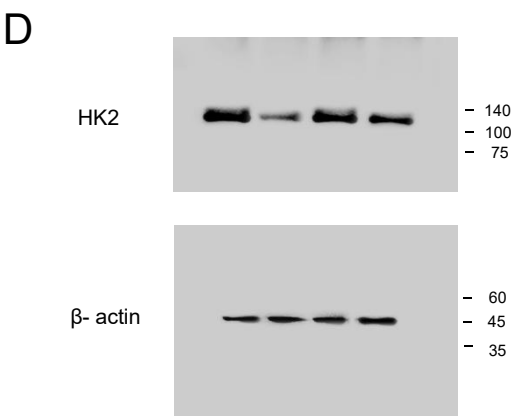

Fig. 2

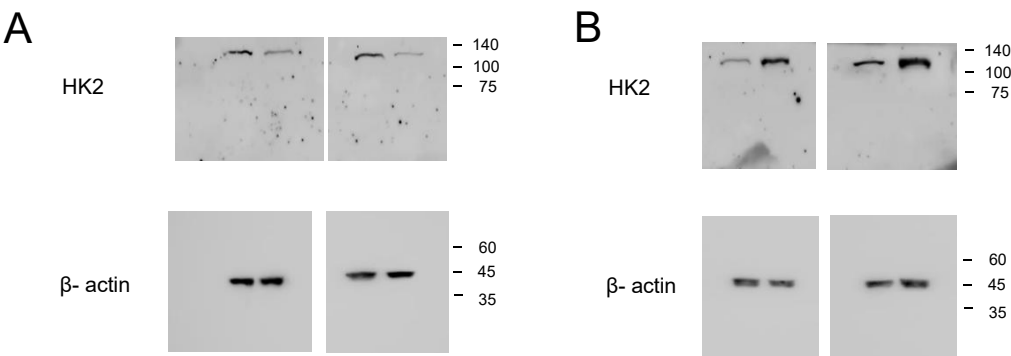

Fig. 3

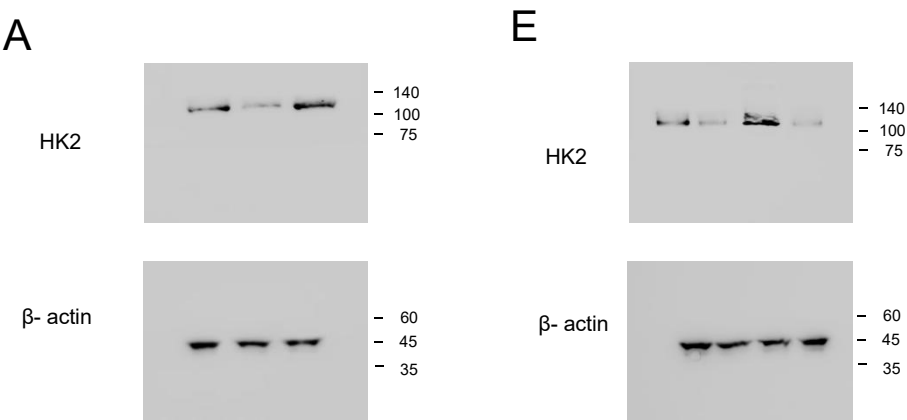

Fig. 4

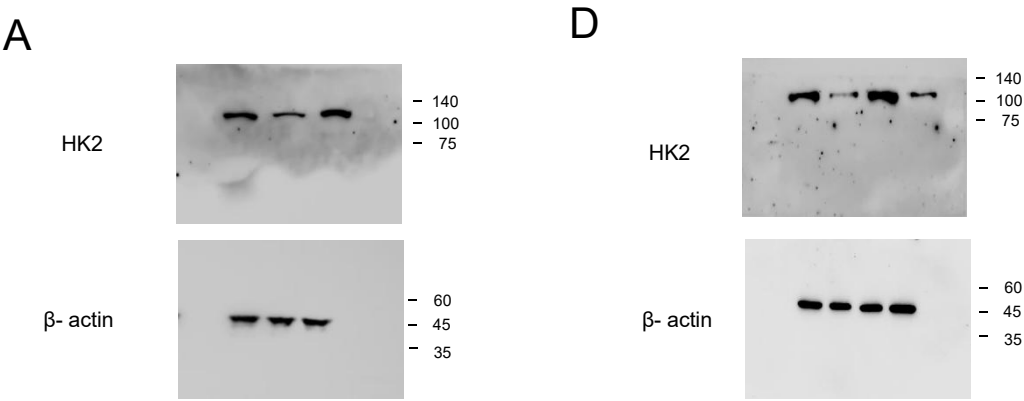

Fig. 6

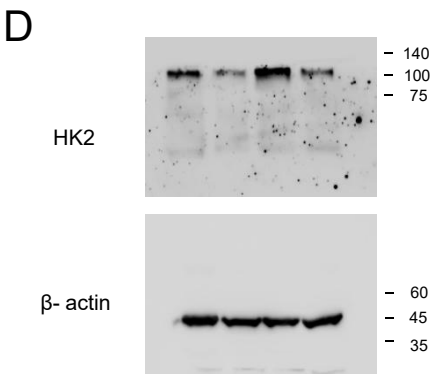

Fig. S1

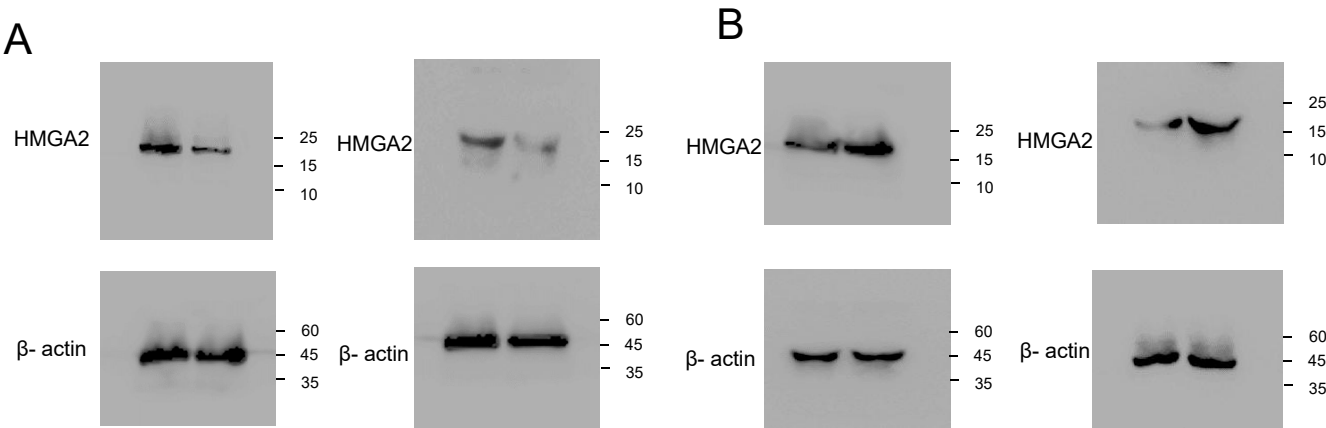

Fig. S2

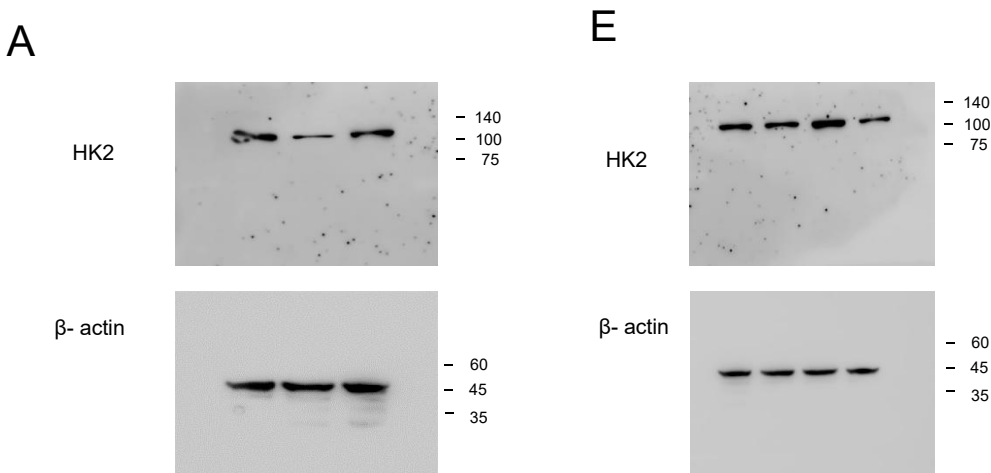

Fig. S3

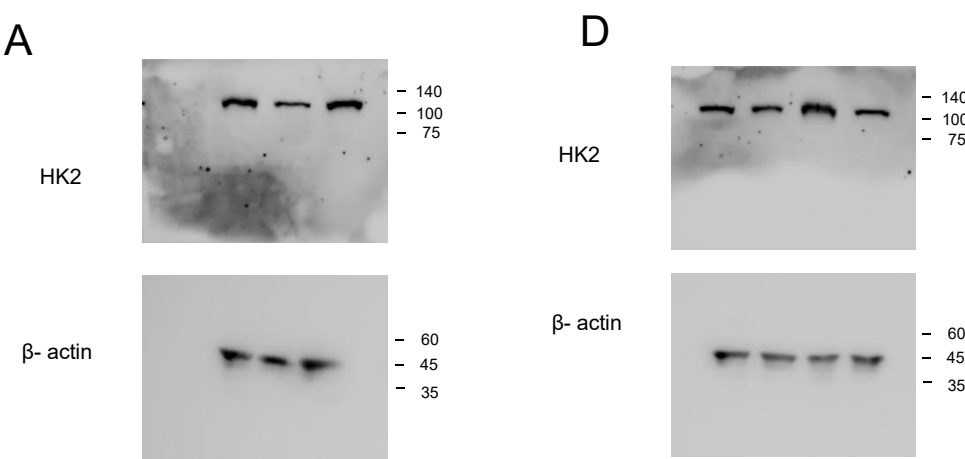

Supplement: Supplementary file 4 — Western Blots Figures [file 41420_2023_1412_MOESM4_ESM.pdf]
