# Supplementary figures and images for: Correction: Let-7b-5p inhibits breast cancer cell growth and metastasis via repression of hexokinase 2-mediated aerobic glycolysis
Source: Cell Death Discov. 2026 Apr 22;12:186. doi: 10.1038/s41420-026-03069-z (PMC13103310; doi:10.1038/s41420-026-03069-z)

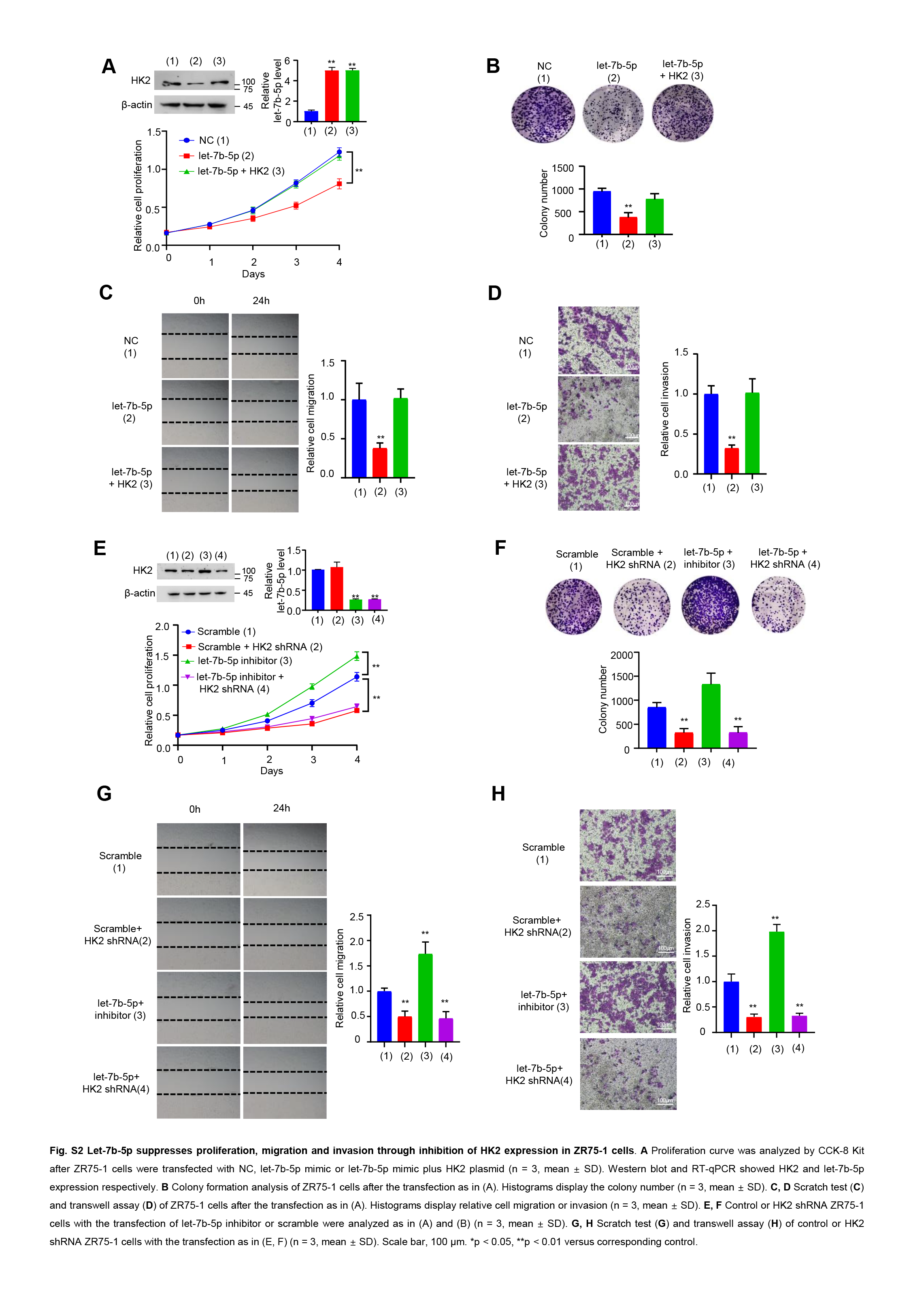

Supplement: Supplementary file 1 — Amended file for figure S2 [file 41420_2026_3069_MOESM1_ESM.tif]

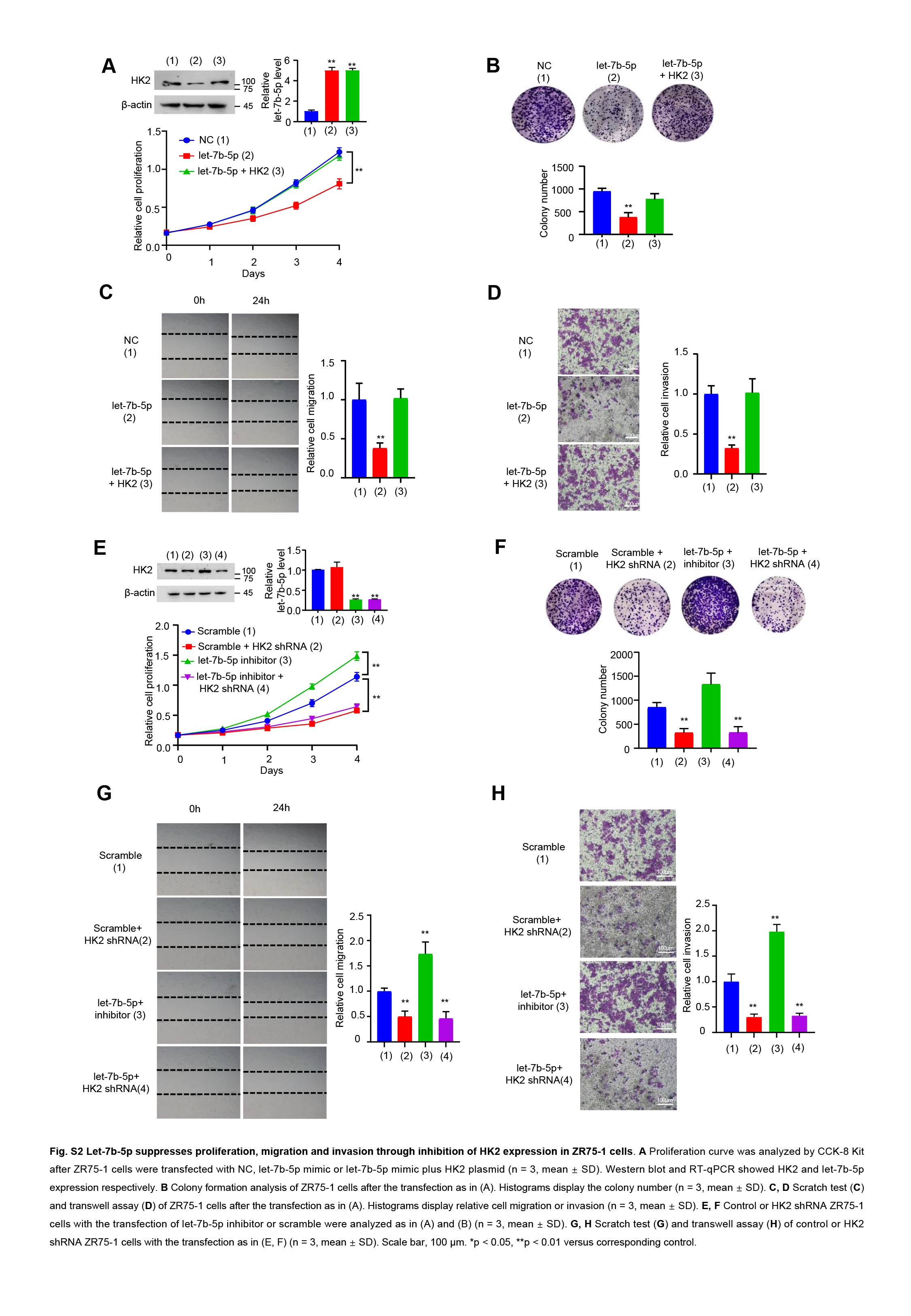

Supplement: Supplementary file 2 — Original file for figure S2 [file 41420_2026_3069_MOESM2_ESM.tif]
